# Psychometric evaluation of the Persian version of the Lymphedema Life Impact Scale (LLIS, version 1) in breast cancer patients

**DOI:** 10.1186/s12955-018-0958-z

**Published:** 2018-06-28

**Authors:** Shahpar Haghighat, Ali Montazeri, Farid Zayeri, Mandana Ebrahimi, Jan Weiss

**Affiliations:** 1 Quality of Life Department, Breast Cancer Research Center, Motamed Cancer Institute, ACECR, Tehran, Iran; 2 Health Metrics Research Center, Institute for Health Sciences Research, ACECR, Tehran, Iran; 3 Proteomics Research Center and Department of Biostatistics, Faculty of Paramedical Sciences, Shahid Beheshti University of Medical Sciences, Tehran, Iran; 4 Lymphedema Clinic, Cox Health Outpatient Rehabilitation, Springfield, Missouri USA

**Keywords:** Lymphedema, Quality of Life, Instrument, Validity, Reliability, Persian, Lymphedema Life Impact Scale

## Abstract

**Background:**

Despite the high prevalence of lymphedema in Iranian breast cancer patients, there is no valid instrument for measuring quality of life in this population. The aim of this study was to assess reliability and validity of the Persian version of Lymphedema Life Impact Scale (LLIS) in breast cancer patients.

**Methods:**

Forward-backward procedure was applied to translate The LLIS from English into Persian. The LLIS is an 18-item measure of physical, psychosocial, and functional impairments caused by lymphedema. Experts and patients assessed content and face validity, respectively. Discriminant validity was evaluated by comparing breast cancer patients with and without lymphedema. Convergent validity was assessed by comparing LLIS score with SF-36 (functional component) and the EORTC-QLQ-C30 (functional component). The construct validity also was evaluated using confirmatory and exploratory factor analyses. Internal consistency was evaluated by Cronbach’s alpha coefficient. Stability was assessed by test-retest analysis over a one-week interval in 13 patients.

**Results:**

In all 446 breast cancer patients were entered into the study. The content and face validity of Persian version of LLIS were acceptable and minor corrections were applied in final version. The questionnaire differentiated well in patients’ with and without lymphedema and not lending support to its discriminant validity. Confirmatory factor analysis showed a good fit for the data. Cronbach’s alpha coefficient in physical, psychosocial and functional subscales were 0.873, 0.854 and 0.884 respectively. Intra-class correlation coefficient of total score of the LLIS was 0.96.

**Conclusions:**

The findings of this study suggest a first indication that reliability and validity of the Persian version of LLIS in patients with breast cancer induced lymphedema was good. Application of this instrument for identifying problems of patients with upper extremity lymphedema may be helpful in designing interventions to improve quality of life.

## Background

The frequency of side effects of breast cancer therapy, such as lymphedema, increases following the increment in survival after breast cancer detection [1]. Lymphedema is a chronic medical condition with excess accumulation of protein-rich fluid in interstitial space of body tissues. It affects activities of daily living, social and interpersonal relationships, and occupational and domestic tasks in 0.84%-21.4% of breast cancer patients [2, 3].It was prevalent in 30% of breast cancer patients in Iranian centers [4]. Lymphedema, a major complication of breast cancer and its treatment, can cause long-term physical and mental health consequences in patients [5]. It may also lead to a number of psychosocial issues including poor body image, reduced confidence in one’s body, physical inactivity, mental disturbances (e.g. anger, sadness, and symptoms of depression), sexual problems, anxiety, and social avoidance [6]. Psychological complications of lymphedema (e.g. depression and social isolation) decrease patients’ abilities and efficacy at work and home [7].

Throughout the literature numerous instruments have been used to measure quality of life (QOL) in patients with lymphedema. Generic QOL measures include the SF-12 [8], the SF-36 [9]. Cancer-related QOL measures include the Functional Assessment of Cancer Therapy (FACT), Functional Living Index-Cancer (FLIC), and European Organization for Research and Treatment of Cancer-Quality of Life Questionnaire (EORTC-QLQ C30) [10]. Lymphedema- specific measures for measuring QOL in either upper limb only, or upper or lower limb are the ULL-27 [11] and LYMQOL [12] respectively. In 2013, Weiss and Daniel considered the advantages and limitations of using two QOL and two functional questionnaires in the lymphedema population, and introduced a new instrument, Lymphedema Life Impact Scale (LLIS) [13]. The total LLIS showed greater than .72 convergent validity with all study questionnaires. Internal consistency, as measured by Cronbach’s alpha coefficient for the total LLIS scale and its subscales were greater than .84. These measures demonstrated the validity of the LLIS in measuring QOL in patients with lymphedema [13]. The LLIS measures physical (pain, heaviness, tightness, strength…), psychosocial (body image, socializing, intimate relations…) and functional (duties at home, duties at work, recreational activities…) impact upon the lives of those with upper or lower extremity lymphedema of either primary or secondary cause.

Despite the high prevalence of lymphedema in Iranian patients following breast cancer treatment [4], there is no specific Persian instrument to measure quality of life and its different aspects in those patients. The purpose of this study is to validate the Persian version of LLIS questionnaire. Providing a valid and reliable instrument in this field can help identify the major problems in breast cancer patients with lymphedema. This understanding may then suggest helpful interventions to decrease impairments and promote quality of life.

## Methods

### The questionnaire

The LLIS was developed by Weiss and validated by Weiss and Daniel in 2015 [13]. The questionnaire consists of three subscales; physical (8 items), psychosocial (4 items) and functional (6 items) impairments. Each item is rated on a five-point Likert scale ranging from 1 to 5, where 1 = no impairment, and 5 = severe impairment. The total score ranges from 18 to 90. The total and subscale scoring were calculated by an Excel "G code" calculator (developer L. Hodgkins, MS, OTR/L, CLT-LANA; Hartford Hospital Rehabilitation Network, Meriden, CT) The “G code” calculator is able to establish a percent impairment from a summed LLIS score, considering missing items. Higher percent of impairment indicates lower quality of life due to lymphedema [13].

### Translation

For purposes of validation of a Persian version of the LLIS, the standard "forward-backward" procedure was applied. Two independent health professionals translated the items from English into Persian and a provisional version was provided. Subsequently it was back translated into English which was approved by instrument designer. After careful cultural adaptation the final version was provided.

Face validity was assessed by 10 patients. Patients judged items on difficulty, ambiguity, and understanding items. Content validity was evaluated by 9 specialists in the field of breast cancer and lymphedema. The experts rated LLIS questions to calculate content validity index (CVI). The CVI is calculated in order to show relevance, clarity and simplicity of each item and involves on a scale from zero to one. It is calculated by rating 0-3 to each item and computing as the number of experts giving a rating 3 to each item, divided by the total number of experts.

In addition content validity ratio (CVR) was calculated in order to quantify the most important and correct content in an instrument. The experts are requested to specify the necessity of each item by scoring them from 1 to 3 with a three-degree range of “not necessary, useful but not essential, essential” respectively. Content validity ratio varies between 1 and -1. The higher score indicates further agreement of members of panel on the necessity of an item in an instrument. The formula of content validity ratio is CVR = (Ne - N/2)/(N/2), in which the Ne is the number of panelists indicating "essential" and N is the total number of panelists. The numeric value of content validity ratio is determined by Lawshe Table. If the CVI was higher than 79 percent, the item will be appropriate and minimum level of acceptable CVR according to Lawshe Table was 0.78 for 9 experts [14]. The mean of CVR and CVI for the LLIS items were 0.93 and .94, respectively. It indicated a strong agreement among the experts about the content validity of questionnaire to measure the problems associated with the condition of lymphedema. One Persian word was nonsense for patients, so recommended changes to the translated questionnaire from experts were applied in development of the final Persian version of LLIS questionnaire.

### Study samples

Patients referred to lymphedema clinic of a Rehabilitation Center and BCRC (Breast Cancer Research Center) Lymphedema Clinic were recruited for this study. All had upper limb unilateral lymphedema due to breast cancer. Comparison group participants were selected from patients without edema referred to those clinics for education or checkup. Some members of the comparison group were selected from the BCRC's follow up clinic. Study participants were recruited between May 2015 and March 2016. Patients with orthopedic problems or psychological disorder history or patients receiving CDT (Complete Decongestive Therapy) were excluded from study. Lymphedema was defined as edema volume difference 200cc or more between both arms.

Four samples were recruited in this study:

1. An initial group of 15 patients with stable lymphedema, undergoing no active treatment were recruited to assess the test-retest reliability of the LLIS in one-week interval. Two patients did not participate in re-test assessment and reliability analysis was achieved on 13 patients.
2. It was necessary to include at least 10 patients per item for providing an acceptable sample size. Considering the 18 items of LLIS questionnaire, a sample of 203 lymphedema patients were recruited at the time of initiating Complete Decongestive Therapy (CDT). Because of incomplete data records, three patients were excluded from study. They included for assessment of construct validity, convergent validity and internal consistency.
3. LLIS scores from a Comparison group of 202 patients were recruited to assess discriminant validity which 200 ones had complete data for analysis.
4. Convergent validity was assessed in a sample of 46 patients with lymphedema by completing LLIS and EORTC QLQ-C30 questionnaires and a sample group of 400 patients who completed LLIS and SF-36 questionnaire. The functional aspects of those questionnaires were assessed for convergent validity. Coefficients of 0.2, 0.4, 0.6, and 0.8 were considered as lower range for weak, moderate, strong and very strong correlation cut points, respectively.

### Additional measures

1. The Short Form Health Survey (SF-36): The SF-36 is a general quality of life instrument that measures eight health related concepts: physical functioning (PF-10 items), role limitations due to physical problems (RP-4 items), bodily pain (BP-2 items), general health perceptions (GH-5 items)), vitality (VT-4 items), social functioning (SF-2 items), role limitations due to emotional problems (RE-3 items), and perceived mental health (MH-5 items). In addition, a single item that provides an indication of perceived change in general health status over a one-year period (health transition) is also included in the SF-36. The instrument is validated in Persian language. [15]. The score on each subscale ranges from 0 (worth condition) to 100 (best condition).
2. The European Organization for Research and Treatment of Cancer Quality of Life Questionnaire (EORTC QLQ-C30): a self-report instrument measuring functioning (physical, role, emotional, cognitive, and social) and physical symptoms and designed to assess the impact of cancer on quality of life. The psychometric properties of the Persian version are well documented [16]. The score on each subscale ranges from 0 to 100. For functioning sub scales the higher scores indicate higher level of functioning whereas for symptom subscale the higher scores indicate greater degree of symptoms.

### Data analysis

IBM SPSS Statistics 22.0 (SPSS, Armonk, NY) was used to compute demographic frequencies and for the reliability/validity analysis. A factor validity analysis was conducted to show whether the LLIS Persian version questionnaire measures or correlates with the theorized construct. For this purpose, a confirmatory factor analysis (CFA) using ESQ software and an exploratory factor analysis (EFA) using SPSS software were performed. Discriminant validity was assessed by comparing the LLIS score, and the LLIS subscales among those with and without lymphedema. It was hypothesized that those with lymphedma would score significantly higher than patients without lymphedema. The students’ t-test was used to compare scores between these two groups. Calculation of the Spearman's correlations coefficient was used for convergent validity when comparing the physical, psychosocial, and functional domains of the LLIS to comparable domains of the SF 36 and EORTC QLQ-C30 questionnaires. The internal consistency of each domain in the LLIS was determined by Cronbach’s alpha. Intra-class correlation coefficients (ICC) were calculated by test-retest analysis. The ICC's level of 0.7 was considered as minimum standard for reliability and above 0.9 as excellent level [17].

## Results

Demographic characteristics of study participants are shown in Table 1. The mean of excised and involved lymph nodes respectively, were higher in patients with lymphedema (12.04, 3.91) compared to the comparison group (10.45, 3.04). The frequency of modified radical mastectomy surgeries in patients with lymphedema compared to Comparisons were 60.9% and 52.7%, respectively. Other patients underwent breast preservation surgery. 76% of lymphedema patients were overweight (BMI > 25), compared to 63% of the Comparison group.

**Table 1:** Clinical and Demographic Characteristics of Participants

Characteristics	Lymphedema patients	Comparison Group	P-value
	Mean (SD)	Mean (SD)	
Age	53.28 (10.95)	51.47 (10.58)	0.093
No of Excised LN	12.04 (6.48)	10.45 (6.15)	0.032
No of Involved LN	3.91 (5.06)	3.04 (3.94)	0.102
Time since cancer diagnosis (months)	42.27 (44.8)	29.7 (31.13)	0.001
	N (%)	N (%)	
Employee	54 (27)	56 (28)	0.911
Education ≥ High school	134 (67.7)	154 (80.6)	0.014
Married	174 (87)	160 (80)	0.079
MRM	109 (60.9)	77 (52.7)	0.145
Receiving Chemotherapy	180 (94.2)	161 (87.5)	0.03
Receiving Radiotherapy	154 (79)	113 (72.9)`	0.207
Underlying disease	71 (35.5)	50 (25)	0.029
BMI >= 25	148 (76.3)	117 (62.6)	0.004

*LN* lymph node, *MRM* modified radical mastectomy, *BMI* body mass index

### Validity

Validity of LLIS Persian version questionnaire was evaluated in three major parts: Factor analysis, discriminant validity and convergent validity.

### Factor analysis

Results of confirmatory factor analysis are summarized in Table 2. The NNFI (Non-Normed Fit Index) and CFI (Comparative Fit Index) were near to 0.9, which is acceptable cut point. RMSEA (Root Mean-square Error of Approximation) value was 0.087, which is higher than acceptable level of 0.05. However its 95% confidence interval was significant. Considering that most indexes yielded from confirmatory factor analyses were near the acceptable level, an exploratory factor analysis was conducted to detect any problems in arrangement of items. Sphericity assumption was assessed by Bartlett's Test. (p<0.0001) Eighteen variables were evaluated by exploratory factor analysis. Three factors had eigenvalue greater than one that jointly accounted for 64% of variance observed (each accounting for 26%, 22.5% and 15.5% of variance observed, respectively). The rotated varimax components are presented in Table 3.

**Table 2:** Factor Validity Evaluation of LLIS Persian Version by Confirmatory Factor Analysis

Fitness Indices	Value of Indices
Bentler-Bonett Normed Fit Index (NFI)	0.856
Bentler-Bonett Non-Normed Fit Index (NNFI)	0.894
Comparative Fit Index (CFI)	0.908
McDonald Fit Index (MFI)	0.909
Root Mean-square Error of Approximation (RMSEA)	0.087
90% Confidence Interval of RMSEA	0.075 – 0.099

**Table 3:** Varimax Rotated of Three Factors of LLIS Questionnaire

	Rotated Component Matrix^a^			
	Component			
	1	2	3	
LLIS1	**0.713**	0.217	0.205	
LLIS2	**0.754**	0.325	0.206	
LLIS3	**0.621**	0.401	-0.006	
LLIS4	**0.772**	0.159	0.114	
LLIS5	**0.884**	0.160	0.047	
LLIS6	**0.726**	0.241	0.409	
LLIS7	**0.635**	0.224	0.430	
LLIS8	-0.011	-0.154	**0.631**	
LLIS9	0.328	**0.651**	0.139	
LLIS10	0.303	**0.821**	0.017	
LLIS11	0.267	**0.813**	0.110	
LLIS12	0.146	**0.759**	0.299	
LLIS13	0.360	0.485	**0.624**	
LLIS14	0.214	0.508	**0.588**	
LLIS15	0.245	0.511	**0.544**	
LLIS16	0.455	0.423	**0.443**	
LLIS17	0.446	0.351	**0.513**	
LLIS18	0.235	0.419	**0.586**	

Extraction Method: Principal Component Analysis

Rotation Method: Varimax with Kaiser Normalization

^a^. Rotation converged in 5 iterations

Data in bold are Statistically significant

### Discriminant validity

The total LLIS score and subscale impairment percentages compared between patients with and without lymphedema to assess discriminant validity. Total LLIS mean impairment in lymphedema patients compared to the comparison group were 38% and 29%, respectively (p<0.0001). Similarly all three subscales of LLIS had higher impairments in patients with lymphedema compared to the comparison group as expected. These differences were significant for physical and functional subscales (p≤0.001) but not for emotional subscale. The results are shown in Table 4.

**Table 4:** Discriminant Validity of LLIS Persian Version Questionnaire

LLIS subscales	Mean % of LLIS score (SD)		P-value
	Lymphedema group (n=200)	Comparison group (n=200)	
Total impairment	38 (22)	29 (24)	<0.0001
Physical impairment	39 (23)	3 (24)	<0.0001
Psychosocial impairment	25 (27)	21 (26)	0.127
Functional impairment	43 (29)	33 (3)	0.001

### Convergent validity

Convergent validity was assessed through comparison of the LLIS and SF 36 questionnaire scores from 400 patients, with LLIS and EORTC QLQ-C30 scores from a sample of 46 patients. (Table 5) All Spearman's correlations coefficients between domains of the LLIS and functional domains of the SF36 and EORTC QLQ C-30 were significant (p < 0.0001).

**Table 5:** Convergent validity of LLIS with SF36 and EORTC QLQ-C30

Percent impairment of LLIS	EORTC QLQ-C30		SF36	
	Role Functioning	Functioning Score (5 subscale)	Physical functional score	(Physical and Social) Functional score
Total LLIS				
r^a^	-0.723	-0.722	-0.457	-0.497
P	<0.0001	<0.0001	<0.0001	<0.0001
Physical LLIS				
r^a^	-0.482	-0.388	-0.402	-0.412
P	<0.0001	<0.0001	<0.0001	<0.0001
Psychosocial LLIS				
r^a^	-0.6	-0.722	-0.344	-0.413
P	<0.0001	<0.0001	<0.0001	<0.0001
Functional LLIS				
r^a^	-0.704	-.715	-0.441	-0.475
P	<0.0001	<0.0001	<0.0001	<0.0001

^a^ r: Spearman correlation coefficient

Findings show that the LLIS total scores correlated higher with the functional scores of EORTC QLQ-C30 (r ≤ -0.72) compared to SF36 (r ≤ -0.457). This higher correlation with EORTC QLQ –C30 also held for all LLIS subscales.

### Reliability

The internal consistency of the questionnaire and its subscales were assessed by Cronbach’s alpha coefficient. The Cronbach’s alpha for physical, psychosocial and functional sub-scales were 0.873, 0.854 and 0.884, respectively. Cronbach's alpha values for patients with and without lymphedema are displayed in Table 6.

**Table 6:** Internal Consistency of LLIS Subscales

LLIS Score	Cronbach’s alpha co-efficient	
	Lymphedema patients (n = 200)	Total patients (n = 400)
Physical Domain	0.853	0.873
Psychosocial Domain	0.852	0.854
Functional Domain	0.879	0.884

The LLIS questionnaire stability was assessed by test-retest analysis. This approach assumes that there is no substantial change in the construct being measured between the two occasions. The test-retest reliability was assessed by administering LLIS twice over a one-week interval. High test-retest reliability level (0.855 to 0.977) in thirteen participants with stable lymphedema was found. (Table 7)

**Table 7:** Test –retest Reliability of LLIS Questionnaire within one week interval

LLIS Score	ICC (n=13)	95% Confidence Interval	P-value
Total impairment	0.962	0.874 - 0.988	<0.0001
Physical impairment	0.927	0.762 – 0.978	<0.0001
Psychosocial impairment	0.855	0.524 – 0.956	0.001
Functional impairment	0.977	0.925 – 0.993	<0.0001

## Discussion

This study demonstrated the validity and reliability of the Persian version of the Lymphedema Life Impact Scale (LLIS). As such, the Persian version of LLIS had a relatively good construct validity and reliability, and thus could be used for measuring quality of life in Iranian patients with lymphedema.

During the psychometric evaluation of the Persian version of the questionnaire, only a few points with minor ambiguity were pointed out by the experts and patients and were corrected accordingly. It seemed that the corrected version had a sufficient clarity in terms of items and content. For higher quantitative accuracy, the CVI and CVR were also measured and the calculated values were at a relatively acceptable level. Although some items had low CVR, they were considered acceptable due to their high mean scores (equal or higher than 2.8 out of 3.00). Weiss [12] assessed the content validity of the same tool by asking for the comments from four experts. Since the mean scores of importance and necessity of each item were greater than or equal to 3 out of 4, (1=not pertinent; 4=highly pertinent), all items were reported acceptable. Although the Iranian medical community has limited knowledge on lymphology, the present study surveyed nine people and obtained good results.

In order to measure the discriminant validity, two age-matched groups with and without lymphedema were evaluated and compared. The two groups had some differences in terms of characteristics and clinical risk factors of lymphedema. The mean of excised lymph nodes (p=0.032) and time interval from cancer diagnosis (p = 0.001) in lymphedema patients was higher than the comparison group. Lymphedema patients were less educated and more obese. The frequency of chemotherapy (P = 0.03) and underlying disease (p = 0,029) were higher in lymphedema patients, too. These findings were concordant with results of previous study about lymphedema risk factors in Iran [4]. The mean total scores of the LLIS in patients with and without lymphedema were 0.38 and 0.29, respectively. In other words, the group with lymphedema perceived 9% higher impairment (P < 0.0001). However, one should be aware that due to a relatively large sample size and low difference of psychosocial subscale between patients with and without lymphedema, the difference of the total LLIS score might be diluted. Differences were also present between the two groups when physical, psychosocial, and functional subscales were compared however, the difference in the psychosocial subscale was not significant. This can be justified by the various individual, family, and social problems faced by patients with breast cancer. So Hyun Lee et al. used the Short Form (SF-36) Health Survey to evaluate the quality of life in patients with cancer-related lymphedema who had surgery at least one year before the study. They found that patients with lymphedema had significant differences with the general population in all subscales of the SF-36 except for vitality and mental health [18]. These findings may indicate that lymphedema has a greater effect on physical and functional aspects of the patients’ quality of life. In fact, these factors need to be examined in future studies. To sum up it is important to note that although here we reported good discriminant validity for the LLIS as pre-hypothesized assumption, this might be challenged on several ground including the fact that we did not indicate how much difference or differences in impairments between patients with and without lymphedema would be satisfactory or acceptable.

The SF-36 was also completed after evaluating the convergent validity of the LLIS in all patients. The relationships between the SF-36 scores and the total scores of the LLIS as well as its physical, mental, and pain subscales were examined in the general population, the group with lymphedema, and the Comparison group. Spearman’s correlation coefficient showed significant inverse correlations in all of the above cases. As described in the Materials and Methods section, higher scores of the LLIS indicated higher impairment in the quality of life and higher scores of SF-36 reflected better quality of life. Therefore, the inverse relationships between the mentioned variables suggested the convergence of the above-mentioned questionnaires. The two groups had significant differences in the mean scores of the physical, somatic pain, and mental health subscales of the SF-36. However, the obtained correlation coefficients in the physical, psychological, and pain subscales of the LLIS in the group with lymphedema did not show very strong relationships. Therefore, it seems that applying SF-36 for the assessment of the quality of life in patients with lymphedema maybe a less than ideal choice. Likewise, Lee [18] and Ahmed [19] failed to find significant relationships between the two groups in most subscales. Rather than using the SF-36, Keeley [12] and Weiss [13] administered the European Organization for Research and Treatment of Cancer Quality of Life Questionnaire (EORTC-QLQ-C30) and found correlation coefficients higher than 0.70 between the LLIS and most subscales of the EORTC-QLQ-C30. In this study the LLIS and EORTC-QLQ-C30 were completed by 46 patients with lymphedema caused by breast cancer and high correlations (> 0.7) were found between the total scores of the LLIS and functional subscales of the EORTC-QLQ-C30. Therefore, the EORTC QLQ C-30 seems to be a more relevant tool in patients with lymphedema than the SF 36, but further studies are required to clarify this finding.

In this study, both confirmatory factor analysis and exploratory factor analysis were used to evaluate factor validity. The acceptable values of the indicators measured in the confirmatory factor analysis, i.e. CFI and RSMA, confirmed the fitness of the 18-item translated questionnaire. However, since some indicators, such as RMSEA, indicated the marginal fit of the model, the researchers suspected that the questionnaire might have a different structure (rather than its original structure arranging items in the physical, psychosocial, and functional subscales) in the population under study in Iran. Exploratory factor analysis was, hence, performed to determine the appropriate arrangement of the items. But according to the results, the item related to infection history was placed in the functional subscale (instead of the physical subscale). Weiss reported the inclusion of an item about the episodes of infection as an advantage of the LLIS over other quality of life tools. The frequency of infections is a factor with undeniable effects on patients’ quality of life and should thus be measured. The LLIS investigates the history of an infection leading to hospitalization or receiving antibiotics in the past two years through a five-choice item scored as 1 to 5 (never, less than once a year, 1-3 times a year, 4-6 times a year, and 7-9 times a year, respectively). In the evaluation of the questionnaire’s reliability, it was observed that this item reduced the internal consistency of the physical subscale. It was, hence, separately scored in another part of the analysis and removed from the physical subscale. This was considered a limitation of the LLIS and would require the classification of the frequency of infection under a different subscale in the future versions of the scale [13]. Similarly, Bogan et al. reported the frequency of infection as a major confounding factor in increasing edema, decreasing mobility and self-esteem, and impairing social communications [20]. However we believe that it would be more suitable to move the infection question from the physical subscale to the functional subscale. The relevance of this argument should, nevertheless, be evaluated by the developers of the LLIS. In contrast one might argue that the overlap among items on factor 2 and 3 raises the question whether they do (partially) measure the same thing. However, continuing to use the current factors seems the most reasonable approach.

In an attempt to measure the reliability of the Persian version of the LLIS, its repeatability over a one-week period was evaluated through test-retest and an intraclass correlation coefficient (ICC) of 0.96 was obtained for the total scores of the scale. The ICCs of the physical, psychosocial, and functional subscales of the LLIS were 0.927, 0.855, and 0.980, respectively. Because of ethical issue for postponing the treatment and acceptable statistical levels of ICC, only 13 patients were recruited for test-retest reliability assessment. However, the original study by Jan Weiss reported the ICCs for the total scores of the LLIS and its subscales as follows: 0.99, 0.97, 0.978, and 0.965, respectively [13]. Thus it is possible to say that the current study showed relatively good stability overtime.

The internal consistency of the LLIS was assessed by calculating Cronbach’s alpha. The obtained values for the total score of the scale and its physical, psychosocial, and functional subscales were 0.873, 0.854, and 0.884, respectively. These values were all at an acceptable level and consistent with the values reported for the English version of the scale (0.85, 0.841, and 0.888, respectively)[13].

The present study focused on the psychometric properties of the LLIS only in patients with lymphedema due to breast cancer and yielded relatively good results. Since the original version of the scale was designed for patients with lymphedema of various etiologies, future studies are recommended to evaluate its validity in different populations of patients with lower limb or primary lymphedema. Use of the LLIS to determine the life impact of lymphedema, can pave the way for interventional studies of methods to improve the quality of life of those living with this disease.

## Conclusion

The findings of this study suggested a relatively good reliability and validity for the Persian version of the LLIS in patients with lymphedema due to breast cancer. However further investigations are needed to achieve stronger psychometric indexes for the questionnaire. Perhaps using this questionnaire in outcome studies might help to improve quality of life in breast cancer patients suffering from lymphedema.
